# Consumer-Resource Dynamics: Quantity, Quality, and Allocation

**DOI:** 10.1371/journal.pone.0014539

**Published:** 2011-01-20

**Authors:** Wayne M. Getz, Norman Owen-Smith

**Affiliations:** 1 Department of Environmental Science, Policy and Management, University of California, Berkeley, California, United States of America; 2 Stellenbosch Institute for Advanced Study (STIAS), Wallenberg Research Centre at Stellenbosch University, Stellenbosch, South Africa; 3 School of Animal, Plant and Environmental Sciences, University of the Witwatersrand, Wits, South Africa; University of Leeds, United Kingdom

## Abstract

**Background:**

The dominant paradigm for modeling the complexities of interacting populations and food webs is a system of coupled ordinary differential equations in which the state of each species, population, or functional trophic group is represented by an aggregated numbers-density or biomass-density variable. Here, using the metaphysiological approach to model consumer-resource interactions, we formulate a two-state paradigm that represents each population or group in a food web in terms of both its quantity and quality.

**Methodology and Principal Findings:**

The formulation includes an allocation function controlling the relative proportion of extracted resources to increasing quantity versus elevating quality. Since lower quality individuals senesce more rapidly than higher quality individuals, an optimal allocation proportion exists and we derive an expression for how this proportion depends on population parameters that determine the senescence rate, the per-capita mortality rate, and the effects of these rates on the dynamics of the quality variable. We demonstrate that oscillations do not arise in our model from quantity-quality interactions alone, but require consumer-resource interactions across trophic levels that can be stabilized through judicious resource allocation strategies. Analysis and simulations provide compelling arguments for the necessity of populations to evolve quality-related dynamics in the form of maternal effects, storage or other appropriate structures. They also indicate that resource allocation switching between investments in abundance versus quality provide a powerful mechanism for promoting the stability of consumer-resource interactions in seasonally forcing environments.

**Conclusions/Significance:**

Our simulations show that physiological inefficiencies associated with this switching can be favored by selection due to the diminished exposure of inefficient consumers to strong oscillations associated with the well-known paradox of enrichment. Also our results demonstrate how allocation switching can explain observed growth patterns in experimental microbial cultures and discuss how our formulation can address questions that cannot be answered using the quantity-only paradigms that currently predominate.

## Introduction

*From time immemorial, man has desired to comprehend the complexity of nature in terms of as few elementary concepts as possible.*

**Abdus Salam**

*Everything should be made as simple as possible, but not simpler*.

**Albert Einstein**

Abdus Salam's observation applies well to the early pioneers of mathematical ecology who between 80–100 years ago used simple coupled nonlinear-differential and difference equations to model the temporal dynamics of interacting biological populations [1], [2], [3], [4], [5], [6] (for reviews see [7] and [8]). Invoking Einstein's dictum, we argue that over the course of time, in the process of developing a comprehensive theory of consumer-resource interactions or, more generally, trophic-flow processes, the models that continue to be invoked are generally too simple to capture some important processes influencing the dynamics of populations. These are processes that relate to a concept of population quality that inter-alia has been articulated in the context of maternal affects (e.g. see [9]), variable C-N ratios and hence palatability of plants [10], and other nutritional or stochiometric measures of an organism's tissue content [10], [11], [12]). All of these affect per-capita growth and death rates, where the latter includes both senescence and susceptibility to exploitation by predators and disease (possibly in both directions when grazers select high quality plants, but it is generally low quality individuals that succumb to predators and disease). Thus the use of a single variable representing merely the abundance of the population, whether as density of numbers or biomass, is often inadequate when processes relating to maternal effects or other quality-related effects are more than secondary in determining population trends. These “carry-over” or “time-delay” processes may sometimes be captured through structuring populations into age or stage classes with their characteristic “time-to-maturation” constants [13], or through explicitly including cohort effects whereby conditions in the year of birth influence subsequent survival and reproductive success [14], maternal effects passed on from mothers to their offspring [9], [15], or other features of the health, body, or nutritional condition of individuals in a population in response to environmental conditions of the recent past [16], [17].

Here we propose that the simplest next step in capturing many of these carry-over effects, without making the details too explicit is to augment the standard “quantity” or abundance variable formulation by adding a second variable to provide a measure of the current “average quality” of each of the populations in a trophic network or food web. The resultant quantity-quality (Q-Q) models are generally simpler than those incorporating three or more demographic classes for each population [7], [18], which is often taken to be the next step in incorporating multiple intraspecific factors into population modeling formulations.

Our Q-Q approach falls within the ambit of second-order dynamical descriptions of population growth. The importance of such second-order descriptions has been advocated for some time, primarily by Ginzburg and collaborators [15], [19], [20]. They take an inertial view of population growth in arguing that environmental forces affect the rate of change of the per-capita growth rate rather than directly affecting the per-capita growth rate itself. This leads them to formulate a second order differential equation for the abundance *N*(*t*) at time *t* involving both 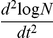 and 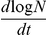, rather than the usual first order equation involving 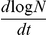 alone. Here we propose an order equivalent mathematical formulation for population growth in positing two first-order differential equations of the process rather than one second-order differential equation.

The model proposed by Ginzburg and Colyvan ([15], p. 90) is highly appealing for its simplicity since it involves only three parameters yet is still able to fit a wide array of population patterns. Their formulation, however, is conceptually too simple in not providing any guidance on how to link consumer-resource equations in a multispecies or food web setting. It also ignores the critical process of explicit switches in the allocation of extracted resources to increasing abundance versus elevating quality. We make explicit this and other key consumer-resource interaction processes: first through the incorporation of an extraction (or feeding) function that appears in both the consumer and resource equations and is at the core of the metaphysiological formulation [21] (also see [22]); and second through an allocation function that distributes varying proportions, depending on season or the state of the populations, of extracted resource to increasing abundance versus average quality of the consumer population.

Ginzburg and Colyvan interpret the second or “hidden variable” in their second order formulation, cast in terms of the derivative of the logarithm of their abundance variable *N*, as a quality variable and interpret it as “energy resources stored inside an individual” ([15], p 44.). We also refer to our second variable as a “quality” variable, but allow a wider interpretation that may differ from one class of organisms to another. In mammals this quality variable may be related to fat storage or other features of individual body condition [16], [23]. In plants it may be related to structural fiber content or carbohydrate storage [24]. In relatively simple organisms, such as bacteria and protists, quality could be related to metabolic potential: the ability to create biomass per unit biomass of the organisms involved as a function of concentrations of environmental nutrients [11] or temperature [25]. Brown, Gillooly et al. [26] have suggested that metabolic potential can be conveniently measured through comparative rates of carbon dioxide uptake in autotrophs or oxygen consumption in aerobes among individuals within populations. The average quality of a population of microbes, as discussed later, may provide a mechanism through which a culture of these organisms switches between quality-dominated versus abundance-dominated growth modes and exhibits growth patterns that cannot be captured by adding time delays to abundance-only models [27].

The dominant paradigm for modeling single and multiple species as dynamical systems has been a demographic one in which, if we ignore migration but explicitly consider extrinsic removal (e.g. predation by carnivores or cropping by herbivores), the rate of change of numbers or density *N_i_* in the *i* ^th^ species (population) has the underpinning structure [28]:
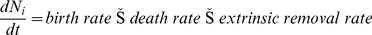
(1)In multispecies contexts where each population is represented by a single quantity or abundance variable, the demographic paradigm dominates in that growth rates are most often interpreted as net birth-minus-death rates, even in the predation and competition models of Lotka and Volterra [2], [6]. Refinements that build on the Lotka-Volterra approach, which itself is neutral on whether to interpret population density in terms of biomass or numbers, remain overwhelmingly dominant today [7], [8], [29].

An alternative paradigm is to think in terms of physiological and extractive process that act directly on the biomass density *X_i_* of the *i* ^th^ species, rather than numbers per se—that is, we do not think in terms of births and deaths—and take a metaphysiological view to obtain the underpinning structure [21], [30], [31], [32], [33], [34], [35]. In this paradigm biomass gains come through feeding and extraction of resources and biomass losses occur in three different ways, which are: an *intrinsic metabolic loss rate*, an *extrinsic decay rate* due to death from senesce and disease, and an *extrinsic removal rate* due to deaths from the consumption of predators or even harvesting by humans. This leads to the model:
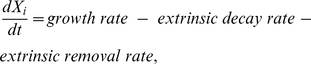
(2)where
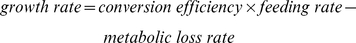
(3)


A demographic paradigm is clearly best suited to addressing problems that focus directly on numbers, such as how best to conserve species close to extinction [36] or how to optimize the number or biomass of animals harvested or culled for food, sport, or some other management related objective [37]. The advantages of a metaphysiological over a demographic paradigm in developing general trophic interaction models has been extensively argued by one of us [21], [22], [31], [38] and its value as a framework for understanding mammalian herbivore ecology has been developed in detail by the other [39]. But like the demographic approach, the application of a metaphysiological multispecies paradigm to developing deep insights into trophic and food web ecology is inadequate because the quality of the biomass of each population itself needs to be known if the potential for this biomass to generate new biomass is to be modeled with sufficient precision. The age- or stage-structure of a population affects its aggregated biomass quality through shifts in the proportion constituted by prime-staged reproductives versus individuals from other stages (e.g. immature or senescing individuals). Improved quality is expressed through lessened susceptibility to mortality from all causes, coupled generally with higher reproductive rates, for most animal populations [40]. The quality of the biomass may also be expressed through the extent of fat stores in animals or carbohydrate stores in plants that restrict the extent of population shrinkage during adverse periods. For plants, however, the features associated with higher biomass quality tend to increase susceptibility to tissue losses via herbivory [41].

Whatever the interpretation of quality, it is clear that growth, conversion efficiency, metabolic expenditures, susceptibility to disease, rates of senescence or other decay processes, as well as predation (extraction), birth and death rates are all dependent in one way or the other on some measure of the quality of the individuals making up the population. The utility of a second-order quantity-quality (Q-Q) versus a first-order quantity-only description of a population's dynamical response, though, depends on the relative time scales over which the quantity and quality variables respond, as measured for example by their “characteristic return times” to equilibrium [42]. If these return or, as we will refer to them, response times of the quantity and quality variables are comparable, as is the case for maternal effects [9] that persist across a generational time scale, then it is essential that the carryover effect of changing biomass quality on the future dynamics of this biomass be taken into account [15], [19], [20]. If the response time for quality is around two orders of magnitude or more shorter than quantity response times (e.g. organisms running out of energy on times scales of days when their generation time is years), then a first order description is likely to be adequate. But if quality response times are at most an order-of-magnitude faster than quantity response times (e.g. organism with storage effects that last months but have generation times scales of a year or two), then a two dimensional description is needed to understand the role that quality may play in shaping the interactions between consumer and underlying resource populations.

A biomass density currency is more generally applicable than a numerical currency in plants and other organisms where individuals are either not distinct or vary enormously in size and the complexity of size class enumeration is best avoided. Nevertheless, the size structure of the population itself affects the intrinsic growth potential and decay rates of biomass because of the allometric scaling of metabolic processes [26],[43]. Representing size, stage or age structure directly, however, leads to models that can become rather complex in multispecies settings, particularly if they are continuous-time integro–differential [44] or partial-differential [45] equation models; although it may be necessary to know details of size or age-class structure in systems where predation (or parasitism) is size or age-class specific [7], [18]. The addition of a single quality variable that represents the changing metabolic action potential inherent in an aggregate biomass measure of the population provides a next level of description. It both is a surrogate for size-distribution effects in those populations where the size distribution changes in a smooth or predictable way throughout a seasonal cycle, and it provides a handle for modeling the response of the population to changes in resource abundance. Finally, a biomass currency is more broadly encompassing than one based on energy flow (e.g. [46], [47]) because biomass includes not only energy, but also mineral and other nutrients needed in some balanced proportions for growth [48], which can be captured at an aggregated level through a concept of average population quality.

In the rest of this paper we formulate our two-state Q-Q approach as a natural extension of the metaphysiological paradigm [21], [22], [31], [38] outlined in the Methods Section at the end of the paper. We then use our Q-Q model to explore aspects of consumer-resource interaction dynamics that cannot be obtained using a one-state metaphysiological approach to modeling each population. In particular, we focus on the role of the extracted-resource allocation function to quantity versus quality in both constant and switching modes in stabilizing consumer-resource interactions. Most importantly we demonstrate that an allocation function, appropriately switching between increasing the abundance versus elevating the average quality of the consumer population can greatly dampen oscillations in consumer abundance that would otherwise be driven by strong seasonal oscillations in the abundance and average quality of the underlying resource populations.

## Results

The development of the model this section extends the metaphysiological Eqs. 4–9 presented in the Methods Section, using notation summarized in Table 1.

**Table 1:** Variables and selected functions and symbols used in models.

*Symbol*	*Description*	*Units or transformation* (*Eqs.#*)	*Range of values*
*t*	independent variable	months or arbitrary time	[0,∞)
*X*(*t*)	consumer pop. abundance	biomass (density) (5,19)	[0,∞)
*R*(*t*)	resource pop. abundance	varies (7,17)	[0,∞)
*Q_X_*(*t*)	avg. quality of consumers	varies (20)	[0,1]
*Q_R_*(*t*)	avg. quality of resources	varies (18)	[0,1]
*x*(*t*)	log population abundance	*x*(*t*) = ln *X*(*t*) (11)	(−∞,∞)
*q*(*t*)	log population quality	*q*(*t*) = ln *Q*(*t*) (13)	(−∞,0]
*f*(*R*,*X*)	extraction function	*R* per (*X*×*R*×*t*) (6)	[0,*δ*]
*g*(*R*,*X*)	per-capita growth rate	*g*(*R*,*X*) = *κf*(*R*,*X*)*R* – *µ* (4)	[–*µ*,*κδ*–*µ*]
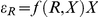	consumer extraction rate	*R* per (*R*×*t*) (9)	[0,∞)
*I*(*x,u,v*), *I*(*R*)	total growth rate	converted *R* per (*R*×*t*) (12, 17)	[0,*κδ*]
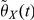	extrinsic decay rate	1/time (8)	[0,∞)
*u*(*t*)	extraction allocation prop.	0≤#<1 (11–13)	0.5 (0–1)
u* = v*	optimal singular allocation	0≤#<1 (15)	0.5 (0–1)
*J*(*u,v*)	optimization criterion	biomass (14)	[0,∞)
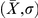	mean and standard deviation of *X*(*t*)	biomass (density)	[0,∞)
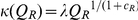	conversion efficiency	# (12)	(0,1)
*ρ* = *R*/*b*	const. resource background	varies (6, 12),	[0,∞)

### A Two-State Q-Q Model with Specified Resources

We begin by considering the growth of a consumer population when resources behave as an aggregated external input *R*(*t*) whose dynamics are independent of the consumer in question. Clearly this is rarely the case, except for plants with a substantial ungrazable biomass component (e.g. underground) or filter feeders in fast flowing streams. It may also be reasonable to ignore coupling consumers back to resources when considering, say, the intra-seasonal dynamics of herbivore quantity and quality variables (i.e. the units of time are days or weeks and the model applies for no more than a couple of months), with feedback coming only when modeling at longer time scales (e.g. seasons or years). Also a particular consumer may only be weakly coupled to a particular resource if it is but one of many consumers on that resource and the consumer itself consumes several different resources. In this case, we may we want to investigate the dynamics of a particular consumer, when the influence of the rest of the food web is characterized in terms of time varying inputs of resource quantity *R*(*t*) and quality *Q_R_*(*t*) variables into the equations describing dynamic changes in the consumer quantity *X*(*t*) and quality *Q_X_*(*t*) variables.

To keep our formulation general, we consider quality to be an index rather than a material measure. For example, if quality relates to optimum storage levels (we mention optimum rather than maximum since it may be that an excess of storage tissue can reduce quality, as is the case of obesity in humans) then it is not the biomass of the storage tissue itself that is the quality variable, because this storage biomass would be included the total biomass quantity variable, but some deviation of storage from the optimum level. Once quality is an index then, without loss of generality, we can constrain it to vary between 0 and 1 and calibrate it so that the growth rate in the abundance of a particular population is maximized when quality is 1 and minimized (i.e. largest negative rate) when quality is 0.

As in [15], we formulate our equations in terms of the logarithmically transformed variables
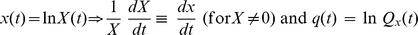
which implies *x*(*t*) ranges over (−∞,∞) and *q*(t)≤0 for all *t*≥0.

In formulating our metaphysiological growth Eq. 5 for a population *X* consuming a resource *R* without any consideration for the quality of the resource or consumer, we introduced a conversion parameter *κ*, a basal metabolism parameter *µ*, a per-capita feeding rate *f*(*R*,*X*)*R*, a decay rate *θ* that includes extrinsic losses from senescence and disease, as well as an extraction rate *ε* on our consumer population *X*. Clearly the quality of both the resource and consumer populations will affect these rates in one way or another.

Perhaps the most obvious effects are that the conversion rate *κ*>0 should be partitioned into a proportion *u*∈[0,1] that is allocated to increasing abundance and a proportion (1-*u*) that is allocated to improving the average quality independent of effects on abundance, and also that the decay rate *θ*>0 should be a decreasing function of the consumer quality variable *q*(*t*). Effects on feeding rates and basal metabolism are likely to be smaller and more complicated (e.g. through size-scaling of metabolic rates). Effects of quality on extraction rate could be large but the direction of the influence (elevating versus depressing quality) could go either way. For example, herbivores may preferentially select relatively high-quality herbage while predators may favor relatively low-quality prey in cases where diminished quality of prey increases their vulnerability because they are weak or sickly. Thus in our formulation, we focus only on the first two effects, leaving the more subtle effects for future consideration.

In formulating the model we assume that under equilibrium conditions an optimal allocation set point *v* exists and that as the optimal allocation u*(*t*) deviates around *v* the allocation process loses some conversion efficiency. This assumption implies that we need to replace *κ* with an appropriate function such as 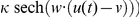, where we remind ourselves that the hyperbolic secant function sech(*y*) has a maximum value of 1 at *y* = 0 and drops off symmetrically on either side of 0. Thus *w* in the expression 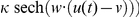 is a scaling factor that controls how rapidly the optimal conversion efficiency *κ* drops off with size of the deviations *u*(*t*)-*v*.

In terms of our second major effect—that is, the decay rate *θ* is a decreasing function of transformed quality *q*(*t*)—we simply posit the simplest possible relationship 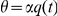 for a senescence rate scaling parameter *α*≥0, where we note that *θ*≤0 because *q*(*t*)≤0 for all *t*≥0. This relationship implies that all individuals are dead by the time their quality index *Q*(*t*) has plummeted to 0, which happens as *q*(*t*)→-∞. The constant *α*≥0 itself can be estimated once we decide how to measure the quality of the population in question or can be fitted based on rates of death in starvation studies, such as the hydra experiments of Lawrence Slobokin (as reported in [19]). In this latter case the quality of hydra relates to its energy content and the presence of symbiotic autotrophic algae able to provide additional energy over time. In the case of plant parts being consumed as a resource, for example, quality may be measured in terms of the amount of indigestible fiber or tannin contents in leaves that are mounting a defense response to herbivory (e.g. as in larch budmoth feeding on Engandine Valley larch: see [49]). In this case, quality *Q* can be scaled so that 1 corresponds to the minimum and 0 to the maximum possible levels of such defensive compounds and structures in consumed plant parts.

Assuming the consumer population is itself not exploited by other populations, then from Eqs. 5 and 10, with the function *I*(*x*,*u*,*v*) defined below, the consumer's quantitative dynamic equation is
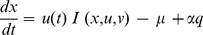
(11)where from Eq. 6 it follows after setting *ρ* = *R*/*b* (assuming *R* constant) and *k* = *K^γ^* that
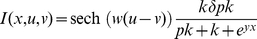
(12)


In deriving the companion equation for the quality variable *q*(*t*), we assume that the rate of increase in quality is proportional to the converted resource intake rate *I*(*x*,*u*,*v*). This proportionality, however, is influenced by the three factors: a general rate constant *α*>0, a factor -*q*(*t*) that ensures *q*(*t*)<0 cannot rise beyond 0 but rather approaches 0 asymptotically (the maximum value *q* can take is 0, which corresponds to *Q* = 1), and a factor 1-*u*(*t*) representing the proportion of resources allocated to increasing quality rather than abundance. Thus the overall rate of increase in quality, before accounting for changes in quality due to removal of individuals through the processes of extraction and senescence, is *a*(-*q*)(1-*u*)*I*(*x,u,v*). The rate of change of *q*(*t*) is also influenced by the two biomass loss process: intrinsic losses due to metabolism at the rate *µ* and extrinsic losses to senescence and disease at the rate *θ* = -*αq*. If we assume that metabolism preferentially draws upon higher-than-average quality biomass (because these contain the greatest concentration of energy or nutrients per unit biomass) with an associated bias *c*>0 per unit metabolism loss rate *µ*, or that senescence preferentially removes lower-than-average quality individuals or ramets from the population with an associated bias rate *b*>0 per unit senescence rate *θ* = −*αq*, then the simplest model that accounts for this is
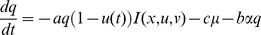
(13)Note that since *q*(*t*)<0, the term -*bαq* (*t*) is positive and hence causes quality to increase, unlike the term -*cµ* which causes quality to decrease.

Eqs. 11–13 may look like they defy Einstein's dictum for simplicity, but they really are simple given that they include the bare minimum needed to account for the processes of: 1.) resource extraction with resource density and intraspecific-density effects, 2.) evolutionarily adapted optimal resource quality-dependent conversion of resource biomass into population biomass, 3.) the consumer's basal metabolism, 4.) consumer population quality-dependent decay from senescence and disease-related deaths (note that the process of predation on the consumer population has not yet been included in this formulation), 5.) the effects of consumer quality itself on its own population growth and decay processes, and 6.) allocation of extracted resources by the consumer to increasing its abundance versus elevating its quality.

With all these processes are included, the population model represented by Eqs. 11–13 has only 11 parameters, several of which in well-studied systems including microcosms [50] can be independently estimated (e.g. *µ*, *δ*, *µ*, and *µ*), with the rest estimated by fitting solution trajectories to population level data.

### Optimal Resource Allocation to Abundance versus Quality

The method of ‘Adaptive Dynamics’ has been developed to assess the equilibrium value (evolutionarily stable strategy) of continuous traits in populations evolving under natural selection that are homogeneous across individuals apart from the values of the traits under selection [51], [52]. These methods, however, are based on maximizing the per-capita growth rate 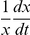 (cf. Eq. 11). In our case, since quality *q* is a feedback that influences the per-capita growth rate, and resource allocation is a strategy that affects both the abundance and quality variables *x* and *q*, with the dynamics of *q* itself dependent on the value of *x*, the method of Adaptive Dynamics cannot be applied directly: a numerical solution is first required that integrates the equations in *x* and *q* (i.e. Eqs. 11 and 12) to be able to evaluate how they impact each other's rates of change. Alternatively an invasion exponent method needs to be applied that accounts for quality-dependent variation in vital rates using evolutionary entropy concepts [53].

Also, as developed more fully below, the most applicable cases arise when the system is subject to seasonal drivers and the optimal strategy involves switching between allocating resources to quality or quantity at different times in the seasonal cycle. In this case, although it may be theoretically possible to switch between *u* = 0 and *u* = 1, physiology will constrain *u* to switch between *u*_min_>0 and *u_max_*<1, where the values of *u*_min_ and *u_max_* are species dependent. Since in this case no equilibrium solution exists, the evolutionarily selected solution will be one that maximizes growth rate integrated over a seasonal cycle or even a full population cycle if oscillating solutions have periods longer than a single season. Additionally, in relatively small populations (i.e. populations containing only hundreds to thousands of individuals), both demographic and correlated environmental stochasticity play an important role in determining rates of extinction. In stochastic systems long-run growth rates are lower than average rates, with the bias increasing with the level of environmental variation [54]. Finally, regarding the question of extinction rates, metapopulation structure becomes important, since small local demes experience extinction at much higher rates than spatially homogeneous populations. In this case the process of Wilsonian deme selection [55] may play an important role in the evolution of an allocation function *u*(*t*)∈[*u*_min_, *u_max_*] in individuals that greatly reduces the risk of extinction of the population over time. The importance of interdemic, however, may be reduced by that fact that the individuals most likely to survive extreme events threatening local population extirpation are those that have allocated sufficient resources towards improving their quality, since these are the individuals that become the founders of population recovery. This issue is complex and will need further investigation.

Assuming some dependence of evolved resource allocation strategies on selection at the deme level, metapopulation processes (average size of demes, movement rates among demes) and resource allocation constraints *u*_min_ and *u_max_* are species dependent. Thus a general understanding of what resource allocation strategies we should expect to see in extant populations, without getting into intricate species specific aspects, can be obtained in the context of a selection of canonical studies. Here we lay out a series of such studies starting with a general optimization framework that provides insight into resource allocation strategies that minimize the probability of local population extirpation under constant environmental conditions. Since we are analyzing the problem in a deterministic framework where extinctions only happen in declining populations, and we know that probabilities of extinction on specified time intervals are inversely related to population size, we consider what strategies maximize the population size. Formally, this question can be mathematically cast in an optimization framework by looking for allocation strategies *u*(*t*)∈[*u*_min_, *u_max_*] for all *t*≥0 that come close to maximizing the value of the integral 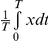. We note that this integral becomes infinitely small (i.e. unbounded below) for any population that goes extinct at some time *t_e_*<T, because as *t* → *t_e_*, *X* → 0 which implies *x* =  ln *X* → −∞. This is precisely the property we want under demic selection where we are interested in solutions that place an infinite penalty in allowing population extinction to occur. Thus a first level of understanding can be obtained by solving the following optimization problem:
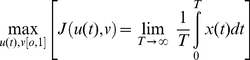
(14)subject to the dynamical Eqs. 11–13.

Fortunately this problem is simple enough to be solved analytically using Pontryagin's necessary conditions [56]. Specifically, in Appendix S1, we show necessary conditions for u*(*t*) and v* to maximize *J*, as defined in Eq. 14, are involve driving the population to an equilibrium solution at which the optimal values are u*(*t*) = v* for all *t*>t*, where
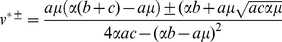
(15)provided *u*_min_≤v*≤*u_max_*. We can use Eq. 15 to assess how v* depends on the various model parameters, although we still need to establish whether v*^+^ or v*^−^ is the solution that maximizes *J* in Eq. 14. Since the conditions are necessary, but not sufficient, it is possible that one of these two candidate solutions might actually locally minimize *J*.

**Figure 1:**
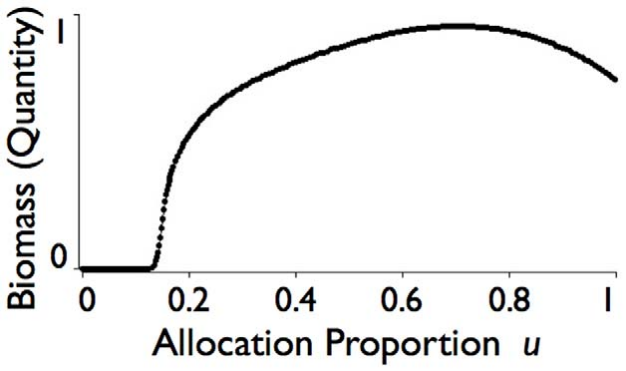
Consumer equilibrium abundance 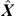 (scale 1 = 30,000 biomass units) is plotted for the baseline set of parameters (**Table 2**) as function of *u* (the proportion of extracted resources extracted that are invested in increasing abundance versus the proportion 1-*u* invested in increasing consumer quality *Q_X_*). Nonzero equilibrium abundance values only occur for *u*≥0.11, and a maximum equilibrium abundance of 28,638 biomass units occurs at *u* = 0.704.

Interestingly, v* is independent of the resource extraction parameters *κ*, *δ*, *ρ*, *γ*, and *k* (i.e. *K*: recall *k* = *K^γ^*) and also of *w* and *T*, although the latter follows from the fact that we are looking at equilibrium solutions satisfying *u* = *v*. The remaining five parameters that Eq. 15 involves are: 1.) The per unit average quality decay or senescence rate scaling parameter *α*>0; 2.) the per-capita metabolic expenditure rate *µ*>0 (from extraction by other species); 3.) the quality improvement factor due to higher-than-average quality biased metabolism *c*>0; 4.) the quality degradation factor due to lower-than-average quality biased senescence *b*≥0; and 5.) the consumer quality resource intake response rate *a*≥0. Since we do not know which root, if either, actually maximizes *J* in Eq. 14, it makes no sense to explore the dependence of v* on these five parameters in the absence of further information. For the set of parameter values (Table 2) that we use as a baseline for the analysis undertaken through numerical simulations presented in later sections, Eq. 15 yields the values v*^+^ = 0.704124 and v*^−^ = 0.295876. Our simulations indicate that u* = v*^+^ = 0.704 (Fig. 1) is indeed the value (to 3 dp in u*) that maximizes *J* under equilibrium constraints. The fact that these solutions match provides mutual co-verification that our analytical and simulation results have been implemented correctly.

**Table 2:** Parameter values and simulation scenarios.

*Param.*	*Role (referring equations)*	*Units* *	*Baseline (& other values used)*
*r*	resource maximum growth rate (22)	1/mnth	0.5 (0.18–0.5))
*a*	consumer quality response param. (14)	1/mnth	0.1 (0–10)
*d_r_*	relative ampl. resource qual. oscillations (24)	0≤#<0.5	0 (0.4, 0.45)
*d_s_*	relative amplitude resource quant. oscillations (23)	0≤#≤0.5	0 (0.4, 0.45)
*δ*	max. resource extraction rate (6)	1/mnth	5 (5.5, 7)
*λ*	max. resource conversion rate (21,6)	0≤#≤1	0.2
*α*	min. consumer quality decay rate is *α/δ* (11)	#	0.1 (0.001)
*µ*	consumer metabolic maintenance rate (4,11)	1/mnth	0.05 (0.01)
*β*	resource level at half max extraction rate (6)	1/mnth	30,000
*γ*	consumer density-dep. abruptness param. (6)	#≥1	2
*K*	consumer density-dep. scaling param. (6)	biomass	10,000
*S*_0_	resource quant. saturation level (23)	biomass	10^6^ (2×10^5^, 5×10^6^)
*c = c_R_ = c_X_*	metabolism higher-than-avg. qual. bias param. (13, 21)	#>0	0.3 (0.6)
*b*	senescence lower-than-avg. qual. bias param. (13)	#>0	0.05
*v*	max. allocation efficiency param. (12)	0≤#<1	0.5
*w*	loss of efficiency when *u*(*t*)≠*v* (12)	#>0	0 (0–5)
*X*_switch_	threshold switching control (25)	biomass	0–30,000
*u*_min_	lower value switching control (25)	0≤#≤1	0–0.5
*u*_max_	lower value switching control (25)	0≤#≤1	0.5-1
*R*(0)	initial quantity of resource	biomass	2×10^5^ (5×10^5^)
*X*(0)	initial quantity of consumer	biomass	10,000 (100)
*Q_X_*(0)	initial quality of consumer	0≤#≤1	0.45 (0.05)

*mnth = time in months, biomass units are arbitrary,

# =  number (either dimensionless or units may be complex to ensure biomass dynamic equations are in units of biomass/m).

In reality population are never at equilibrium. First, we can expect stochastic influences to continuously perturb population size. Second, populations are influenced by seasonal, annual, and multiyear oscillations in environmental conditions. Third, some population processes are intrinsically oscillatory through delayed feedbacks. We explore questions relating to these latter two aspects in the rest of this paper, leaving an investigation of stochastic aspects for future studies.

### Quality and Oscillations

As reviewed by Turchin [8] investigations into the causes of oscillations in biological population began with Charles Elton's work on fluctuations in the abundance of Norwegian lemming, Canadian lynx, and British vole populations. The primary causes are thought to derive from consumer-resource interactions [8] driven, to some extent, by seasonal cycles; although Ginzburg and collaborators have argued for the importance of maternal effects in producing oscillations [9], [15], [57].

It is a well-known mathematical fact that first-order autonomous differential equations cannot oscillate. In such equations oscillations require that explicit time delays [58] be incorporated or that the equations be elaborated to either a first-order, nonlinear, discrete-time formulation (i.e. a first-order nonlinear difference equation—see [59] or [60]) or to a second-order system of differential equations. Second order systems arise when modeling consumer-resource interactions (see [8] and the references therein) or through the incorporation of inertial terms into a Newtonian-type formulation of population growth ([15], [20]). In difference-equation formulations we need to take care not to artificially induce oscillations of spurious period through an arbitrary choice of step size. Rather, step size should be selected to reflect generational processes, such as maternal effects, or seasonal processes (e.g. [61]) to reflect annual cycles in temperature and precipitation and the impacts these cycles have on the abundance and quality of biological resources that drive the dynamics of population exploiting these resources.

An interest in population fluctuations naturally leads to the question of the extent to which quantity-quality dynamics in-of-themselves induce oscillations in populations and what the periodicity of such oscillations would be. Since we know that seasonality can induce oscillations, as can consumer-resource interactions, we can only answer this question by separating out these various causes by considering the inherent ability of our quantity-quality formulation to induce oscillations in the absence of seasonal drivers and the coupling of populations to their resources at a lower trophic levels or their consumers at higher trophic levels. Thus we address the question in the specific context of oscillations in Eqs. 11–13 as a function of different parameter values, noting that the value of *ρ*, which we recall is given by *ρ* = *R*/*b* (cf. Eq. 6 and 12), represents the underlying, but constant in this case, resource level that the population extracts for growth in the abundance measure *x* and average quality measure *q*. Furthermore, since this analysis necessarily assumes that all parameters are constant (i.e. seasonal drivers and other time-varying drivers are absent—such models are said to be autonomous), we assume *u*(t) is constant and, without loss of generality, select *u* = *v*∈(0,1). Further we note that the points 0 and 1 are not included in this range since *u* = 0 implies *q*(*t*)→−∞ and *u* = 1 implies *x*(*t*)→ −∞ as *t*→∞. In short, our focus is on the existence and stability of a finite equilibrium solution 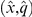 to Eq. 11–13 for constant *u*.

In Appendix S2, we demonstrate that our Q-Q formulation does not produce oscillations when the background resources are fixed. This may seem a surprising result in light of the existence of well-described maternal effects leading to oscillations. However, in real populations background resources are never constant, unless very carefully controlled experimental approaches are taken to ensure such constancy. Thus, for example, colonies growing in Petri dishes or populations growing in well-mixed containers draw down resources if the resources are not replenished through a constant resource input, such as in the experiment of [62]. The fact that our Q-Q model does not produce oscillations for populations embedded in a constant environmental (i.e. resource) background suggests that in real systems sustained oscillations arise either from through consumer-resource couplings, of which the Lotka-Volterra predation model [2], [6] is the best known theoretical example, or through oscillatory forcing by underlying environmental drivers. In practice, of course, we typically see a complex combination of several consumer-resource interactions mutually intertwined through generalist feeding patterns and coupled with environmental forcing at several different frequencies (diurnal, lunar, seasonal, solar and earth's orbit and spin-inclination cycles).

It is worth reinforcing here that discrete-time equations cannot properly account for the effects that fluctuating environmental drivers have on demographic and ecological process if the cycles have periods less than size of the time step underpinning the equations. This statement applies equally if the quality of individuals is incorporated and that quality again exhibits changes in values over time scales smaller than the time step used to formulate the equation (e.g. if quality varies over seasons and the step size is annual, or if the quality varies across years and the step size is generational for organisms that live many years).

Differential equation formulations, by virtue of their continuity in time, avoid the pitfall arising from an inappropriate choice of model iteration step size, when addressing questions relating to the biological interpretation of characteristic oscillation frequencies associated with the model; although it behooves the analyst to ensure that the algorithms used to simulate the equations are numerically well-behaved (i.e. converge to the real solution as the simulation step-size decreases). In our simulations below, we first investigate how consumer abundance is impacted by the value of the allocation proportion parameter *u*(*t*) when assumed constant and set to *v* so that extraction function expressed in Eq. 12 is now independent of *u* and *v* and reduces to
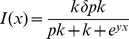
(16)


We then consider how this resource extraction allocation function, switching between growth in consumer abundance (*u*(*t*) = 0) and elevation of quality (*u*(*t*) = 1), can stabilize population fluctuations, including if the switching is incomplete: i.e. *u*(*t*) switches between *u*_min_ and *u*_max_, where 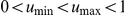.

### Consumer-Resource Interactions

To obtain new insights into factors influencing the period and amplitude of oscillating populations, we begin by extending the metaphysiological consumer-resource model, represented by Eqs. 7, to our Q-Q framework modeled by Eqs. 11–13. After that we explore the oscillatory behavior of a simplified version of this four-dimensional model under the assumption of seasonal drivers underlying resource abundance and quality.

In our metaphysiological Q-Q framework, denoting the actual (i.e. not logarithmic) abundance of the resource by *R* and that of the consumer by *X*, and their untransformed quality indices by *Q_R_* and *Q_X_* respectively, the interactions are described by the four equations (Table 1)
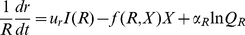
(17)

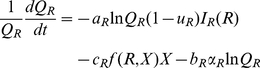
(18)

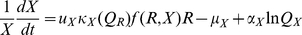
(19)

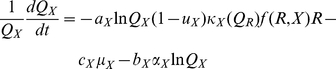
(20)where *I*(*R*) expressed in Eq. 16 is the resource population's per-capita extraction rate (*R* is a photon or nutrient flux if the resource is a plant or is a plant population if the resource is a herbivore), *f*(*X*,*R*) expressed in Eq. 6 is the consumer population's per-capita per-unit resource feeding-rate function, and *µ_X_* is the rate at which exploiters expend biomass to meet metabolic needs.

In our formulation of Eqs. 11–13, we mentioned that the conversion rate *κ*>0 should be a decreasing function of resource quality *Q_R_* in the case of herbivory since, for example, lower quality plants are those that are either defended by chemical compounds that the consumer needs to detoxify or through inclusion of indigestible fiber in leaves and other grazed parts of the plant. A relatively simple function that accounts for the bias that extracted resources will have a higher-than-average quality than the resource population itself, and is dependent on the senescence rate bias parameter *c_R_*>0, is
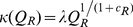
(21)where 0<*λ*<1. To verify that this form has the desired properties we note that: i.) for any *c_R_*>0 the resource quality has at its theoretical (i.e. not realized in practice) maximum *κ*(1) = *λ* when *Q_R_* = 1 and its theoretical minimum *κ*(0) = 0 when *Q_R_* = 0; ii.) the function is linear in *Q_R_* for *c_R_* = 0 (i.e. when there is no bias in the senescence rate as function of quality) and iii.) the function is increasingly super-linear in *c_R_*—i.e. 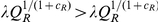 for any 0<*Q_R_*<1 whenever *c*_1_>*c*_2_—so that the quality extracted is increasingly higher than average with increasing *c*.

### Seasonal Drivers

As a first step to understanding the dynamics of a consumer exploiting a resource that varies seasonally in both abundance and quality, we simplify Eqs. 17 and 18 as follows. First, we assume the resource grows logistically, driven by a seasonally varying carrying capacity *S*(*t*)—i.e. in Eq. 17 we make the substitution
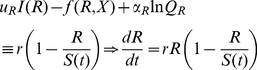
(22)where, assuming the units of time are months, we set
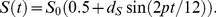
(23)This latter form implies that the parameter 0≤*d_R_*<0.5 determines the amplitude of *S*(*t*)>0 around its average value *S*_0_. Second, we remove Eq. 18 for the quality of the resource and replace it with the periodic input function
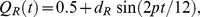
(24)where 0≤*d_R_*≤0.5 ensures that 0≤*Q_X_*≤1, and has an average value of 0.5.

In this case Eqs. 17–20 reduce to a system of three equations containing the following time constants, characterizing five key processes that influence the period and amplitude of oscillations when they emerge as a result of the consumer-resource interaction process:

1. A maximum resource per-capita growth rate *r*: this rate occurs at densities *R*(*t*) well below the carrying capacity *S*(*t*), with a concomitant per-capita decline rate of negative *r* that occurs at double the carrying capacity density (this can arise if the carrying capacity *S*(*t*) drops considerably through its seasonal cycle).
2. A maximum resource extraction rate *δ*: this rate is reduced by the resource quality variable *Q_R_*(*t*), which we expect to average around 0.5 in our simulations, and is also reduced by a normalized functional response to resource and consumer densities *F = f*(*R*,*X*)*R/δ*, which satisfies 0≤*F*<1 (*F* is 1 when resources are large and consumers are not too large, and close to 0 when resources are low or consumer-to-resource density ratio is relatively large).
3. A maximum consumer rate of increase *λδ*: (cf. Eq. 21) this rate is *λ* times the resource extraction rate and so is also modified by the current value of *F*.
4. A maximum consumer rate of decay (*m* = *µ* - *α* ln *Q_X_*): although this rate may typically be in the range [*µ*,*µ*+10*α*] (note: ln *Q_X_*<0), it can rise without bound if consumer quality plummets. In doing so, it will then rapidly drive the population to 0 as individuals become starved of resources.
5. A consumer quality response rate *a*: this rate scales the response of the consumer quality variable to changes in the resource extraction rate, but is moderated by how close the quality variable is to 1 with the rate of approach to 1 declining linearly with distance from 1. It also scales the rate of increase in quality due to preferential removal of low quality individuals during the decay process, with a scaling constant *b* determining the extent to which decay acts differentially on low quality individuals. Also together with a second scaling constant *c*, the parameter *a* determines the maximum rate at which quality declines when at its maximum value of *Q_X_* = 1.

In our analysis we select a baseline set of population parameter values (Table 2) and then explore the impact of introducing seasonal drivers (by making parameters *d_S_* and *d_R_* in Eqs. 23 and 24 non-zero), changing the response-time constants of the resource (contrasting values of the parameter *r*) and consumer quality (contrasting values of the parameter *a*) equations, as well as perturbing the maximum value of the resource extraction rate (contrasting values of the parameter *δ*). Most importantly, however, we also explore the effects of different investment rates *u* in the relative proportion of resources that are allocated to increasing population abundance versus elevating the average quality of the population under the simplifying assumption that *u*(*t*) = *v*, where as discussed earlier (cf. Eq. 12) *v* is assumed to be the most physiologically efficient value of *u* over the long term. In a final simulation, we explore aspects of allowing *u*(*t*) to respond to seasonal changes in population abundance.

Our analysis is conducted through numerical simulations used to explore contrasting values in the above rate parameters on consumer-resource dynamics in the context of population oscillations and possible population collapse, as well as the extent to which the period of oscillation is intrinsic to the system or influenced by seasonality in the resource carrying capacity *S*(*t*) and resource quality *Q_R_*(*t*). For generality and simplicity, we will not specify the units of biomass other than to assume the units of *X*(*t*) and *R*(*t*) are in the same units, noting that the parameters *K* and *S*_0_ scale the equilibrium levels of the consumer and resource populations respectively in these same units. All rate parameters are in units of inverse months (1/mnth) so that if a rate parameter has a value of 0.5, then after 1 month it will have caused the population to increase to approximately *e* ^0.5^ = 1.65 (165%) or decrease to approximately *e* ^−0.5^ = 0.61 (61%). Conversely, if an individual consumes 5% of its body weight per day, which is 150% of its body weight per month, then the per-capita instantaneous rate of resource consumption is ln(1.5) = 0.41. The doubling (parameters associated with increases) or halving (parameters associated with decreases) time of any of our variables under the influence of a parameter *p* is calculated using the equation *t* = ln2/*p*, which is a convenient way to characterize the response time of a rate parameter.

**Figure 2:**
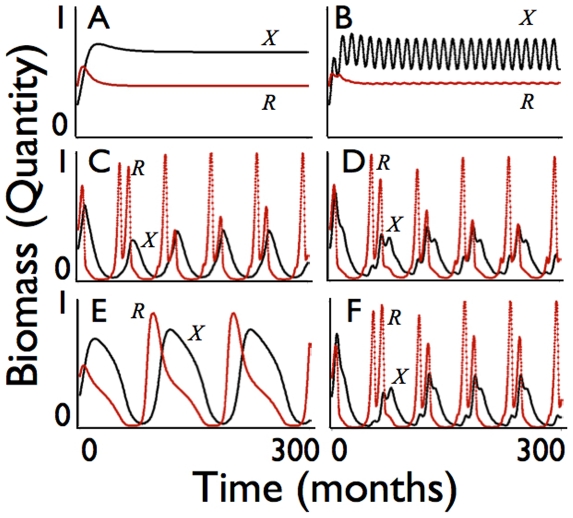
Consumer abundance *X*(*t*) (black: scale 1 = 40,000 biomass units) and resource abundance *R*(*t*) (red: scale 1 = 500,000 units) are plotted over 300 months for the set of baseline parameters listed in **Table 1** for the cases of periodic environmental forcing: **A.** no forcing (*d_R_* = 0.0, *d_S_* = 0.0); **B.** resource quality forcing (*d_R_* = 0.45, *d_S_* = 0.0); **C.** resource abundance forcing *S*(*t*) (*d_R_* = 0.0, *d_S_* = 0.45); **D.** resource quality and abundance forcing (*d_R_* = 0.45, *d_S_* = 0.45); **E.** parameters as in A. except *δ* has been increased from 5 to 5.5; **F.** parameters as in E. except seasonal forcing (*d_R_* = 0.45, *d_S_* = 0.45) has been added.

Numerical simulation of Eqs. 19–24, using the baseline set of parameter values, reveal that in a constant resource environment (i.e. *d_R_* = 0.0 and *d_S_* = 0.0) population abundance equilibrates for all constant values of the extraction rate allocation proportion *u* within the range [0,1] (Fig. 1; a typical trajectory is given in Fig. 2A). The equilibrium values 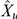 so obtained, however, are only non-zero (in effect exceed the cutoff threshold of 1) for *u*>0.11, achieving a maximum value of 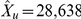 at *u* = 0.704. As we previously mentioned, this is the value obtained by substituting the model parameter values in the expression given by Eq. 15, even though this equation was derived for the two-dimensional system modeled by Eqs. 11–13, while the values in Fig. 1 are derived from numerically simulating the long-term behavior of the higher dimensional model represented by Eqs. 19–24. The reason for the equivalence is that the optimal investment proportion given by Eq. 15 does not depend on parameters in the extraction function Eq. 12, so that in the absence of seasonal drivers in Eq. 22–24 (i.e. *d_R_* = 0.0 and *d_S_* = 0.0) the equilibrium consumer abundance levels in both systems, though different because the resource levels supporting the consumers in the two models are different, will be maximized by the same value v*^+^ whenever the remaining parameters *a*, *b*, c, *α* and *µ* are the same in both models. Again we stress, because the issue of verification is so central to the confidence we can place in our numerical results, that the agreement of values computed in two completely independent ways provides mutual co-verification for the mathematical correctness of our analytical expressions and for the computer code used to generate our numerical results.

The approach of both the resource and consumer solutions to equilibrium values (Fig. 2A), used to produce Fig. 1, is lost once environmental forcing is included in the resource equations. We now consider the impact of seasonal forcing on the abundance of the consumer and its feedback on the oscillating resources. First we consider the case when only the quality of the resource oscillates over a 9-fold range of values (Fig. 2B. *d_R_* = 0.45, *d_S_* = 0.0). In this case the behavior is rather regular with the seasonal forcing of resource quality producing relatively small oscillations on the abundance of the consumer that then feedback to produce even smaller oscillations on the abundance of the resource. When the resource abundance itself is made to oscillate over a 9-fold range around its baseline value while resource quality is kept at its baseline value, then the abundance of the consumer begins to show strong oscillations that are amplified through feedback with a significant drop in the average consumer and resource values over the 25-year (300-month) simulation interval (Fig. 2C. *d_R_* = 0.0, *d_S_* = 0.45). The frequency of these oscillations is approximately 1/5 per year (implying a period of 5 years). If a 9-fold resource quality oscillation is now imposed on top of the 9-fold resource abundance oscillations, the 1/5 frequency and large amplitude of the consumer oscillations dominate, but now with a small amplitude wave of frequency 1—i.e. the annual frequency of the resource quality oscillations—imprinted upon it (Fig. 2D *d_R_* = 0.45, *d_S_* = 0.45).

The size of the oscillations, the shape of the transients, and even the frequency of the dominant oscillations appearing in Fig. 2 are rather sensitive to the different relative values of the time constants associated with the five key processes listed above (Fig. 2E–F). For example, in the absence of seasonal forcing when the maximum extraction rate *δ* is increased from 5 to 5.5, the equilibrium is lost and a cycle of period 8-years emerges (Fig. 2E, *δ* = 5.5, *d_R_* = 0.0, *d_S_* = 0.0). Interestingly, if seasonal forcing is now reintroduced (Fig. 2F, *δ* = 5.5, *d_R_* = 0.45, *d_S_* = 0.45) the period 8 oscillations are lost and the period 5 oscillations return, indicating how the emergent oscillations have periods that are nonlinearly dependent on underlying population process rates and seasonal drivers.

### Periods and Relative Rates

By changing the relative values of the different rate constants listed above, all kinds of behavior can be induced in the abundance of consumers, from extinction, through the existence of a positive stable equilibrium, to stable oscillations with a range of periods. As observed, though, by Murdoch et al. [63] and elucidated by Ginzburg and Colyvan [15], consumers specializing on a single resource are unlikely to oscillate with periods less than 6, unless driven by seasonal drivers, in which case the oscillations may collapse to period 1, the period of the seasonal cycle. Our model exhibits this same behavior (Table 3), as we vary the resource response rate parameter *r* and the consumer quality response rate parameter *a*, with the remaining parameters at their baseline values (Table 2) in both constant (*d_R_* = 0.0, *d_S_* = 0.0) and seasonally forced (*d_R_* = 0.45, *d_S_* = 0.45) backgrounds. In the constant background, the consumer-resource interaction supports a stable equilibrium (Fig. 2A) for the baseline parameters, but as the resource response rate decreases (Table 3A, Case 1) from *r* = 0.5 in steps of 0.01, oscillations set in at *r* = 0.48 with the rather long period of approximately 18 years. This drops to a minimum period of around 8 years for *r* around 0.40 to 0.35 and starts to increase up to a period of approximately 12 years at *r* = 0.21. For *r*≤2.0 the consumer population goes extinct. If seasonal forcing is added (Table 3A, Case 2) then the period is smallest at just under 5 years for the fastest resource response rate considered (*r* = 0.5) rising to around 11 years at *r* = 0.19, but going extinct for *r*≤0.18.

**Table 3:** Period of consumer-resource oscillations for selected values of the resource response rate *r* (**A**.) and the consumer quality response rate *a* (**B**.), with the remaining parameters at their baseline values (Table 2) except as noted.

Parameter	Case 1	Case 2
**A**.: *r*	No seasonality*d_R_* = 0 and *d_S_* = 0	Seasonal Forcing*d_R_* = 0.45 and *d_S_* = 0.45
0.5*	Equilibrium	Period ∼4.8
0.49	Equilibrium	Period ∼4.9
0.48	Period ∼18	Period ∼4.9
0.47	Period ∼11	Period ∼5.0
0.45	Period ∼9	Period ∼5.1
0.40	Period ∼8	Period ∼5.7
0.35	Period ∼8	Period ∼6.2
0.30	Period ∼9	Period ∼7.2
0.25	Period ∼10	Period ∼8.3
0.21	Period ∼12	Period ∼10
0.20	Extinction	Period ∼10
0.19	Extinction	Period ∼11
0.18	Extinction	Extinction
**B**.: *a*	Baseline response *r* = 0.5*d_R_* = 0.45 and *d_S_* = 0.45	Rapid response *r* = 0.3*d_R_* = 0.45 and *d_S_* = 0.45
0.00	Extinction	Extinction
0.02	Period 1	Period 1
0.03	Period 1	Period ∼5.6
0.05	Period 1	Period ∼6.3
0.06	Period ∼4.3	Period ∼6.7
0.08	Period ∼4.7	Period ∼6.9
0.10	Period ∼5.0	Period ∼7.1
0.15	Period ∼5.1	Period ∼7.9
0.25	Period ∼5.4	Period ∼8.7
0.50	Period ∼6.0	Period ∼9.5
1.00	Period ∼6.8	Extinction
10.0	Period ∼7.2	Extinction

*solution is illustrated in Fig. 2A.

The question of how the periods of oscillations arising from consumer-resource interactions are influenced by various rate constants in the model can certainly be addressed using current Lotka-Volterra-like and other first-order species model paradigms, including discrete-time paradigms; e.g. as discussed by Murdoch et al. [7]. However, the question of how the average quality of a population will impact such oscillations cannot even be asked using these approaches, but requires a Q-Q paradigm of the type formulated here. We addressed this question using Eqs. 19–24 (Table 3B). Our analysis indicates that when the quality response rate to resource intake is 0, the population goes extinct because quality asymptotically approaches 0. For non-zero quality response rates *a*>0, relatively small (i.e. slow) response times have little effect and abundance oscillates with its seasonal drivers—i.e. the period is 1. As the quality variable comes into play with increasing responses times *a*, so the period begins to increase. For the case *r* = 0.5 (Table 3B, Case 1) the period jumps from 1 to 4.3 at *a* = 0.06 and then increase steadily to cap out at around 8 years. For the case *r* = 0.3 (Table 3B, Case 2) the period jumps from 1 to 5.6 at *a* = 0.03 and then steadily increases to cap out at around 10 years, though unlike the case *r* = 0.5 the consumer goes extinct for *a*≥0.77 (not shown).

### Allocation Switching and Stabilization

The fact that quality has an influence on the period of oscillations that arise from consumer-resource interactions raises the question of the extent to which individuals can dampen or alter the period of consumer-resource oscillations by manipulating the proportion of resources over time that they allocate to growth in abundance versus elevating the average quality in the population. The most extreme version of this type of manipulation is to switch back and forth between *u*(*t*) = 0 (all extracted resources allocated to elevating the average quality of individuals in the population) and *u*(*t*) = 1 (all extracted resources allocated to increasing population abundance). In the context of maximizing *J* defined in Eq. 14, solutions that switch between lower and upper bounds are called “bang-bang” and are known to be optimal when the problem is linear in the “control” function *u*(*t*), though so-called singular control components, where *u*(*t*) = *v* and *v* is a constant that lies between 0 and 1, also play a role in the optimal solution over a central segment of the interval [0,T] [56].

For systems that are not fully described by the equations used to model their dynamics (in our case Eqs 17–20 are only an approximate description of the processes driving change in the variables of interest) and for systems that are subject to stochastic perturbations, an “open-loop solution” to a formulated deterministic maximization problem, as in encapsulated in our Eq. 14, is moot. More appropriate are “feedback or adaptive solutions” that self-correct when the model strays from reality: such solutions posit explicit explanations of how organisms have evolved to respond to change that is not completely predictable [64]. Thus rather than solve for the optimal solution that corresponds to our specific set of baseline parameters (which themselves are of no special significance), we explore how feedback rules based on the state of the variables perform in stabilizing population fluctuations. As recently hypothesized and demonstrated by Ginzburg et al. (in review) in the context of discrete time models, populations appear to have evolved to avoid the large fluctuations, because populations are most vulnerable to extinction every time they pass through a trough of a large amplitude oscillation.

**Figure 3:**
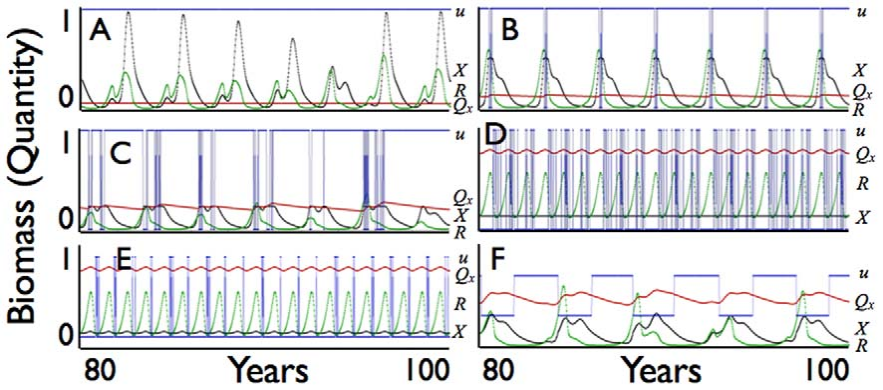
The trajectories of consumer abundance *X*(*t*) (black: scale 1 = 30,000 units) and quality *Q_X_* (red: scale 0–1), resource abundance *R* (green: scale 1 = 600,000) units) and resource extraction allocation function *u*(*t*) (blue: scale 0–1) given by Eq. 25 are plotted over years 80–100 (to avoid effects of initial conditions) of a simulation driven by strong season fluctuations in resource carrying capacity and quality (*d_R_* = 0.45, *d_S_* = 0.45) for the baseline parameters, except here *δ* = 7 and *X*_switch_ varies in steps of 100, for the following cases, with statistics for *X*(*t*) (min, max, mean, square-root of variance) calculated over years 80–100 in parenthesis: **A.** *u*_min_ = 0, *u*_max_ = 1, *X*_switch_ = 29,300 (*X*_min_ = 410, *X*_max_ = 29292, 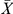 = 5486, *σ* = 7541); **B.** *u*_min_ = 0, *u*_max_ = 1, *X*_switch_ = 15,000 (*X*_min_ = 295, *X*_max_ = 15054, 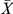 = 5035, *σ* = 4990); **C.** *u*_min_ = 0, *u*_max_ = 1, *X*_switch_ = 7,000 (*X*_min_ = 384, *X*_max_ = 7049, 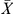 = 3881, *σ* = 2520); **D.** *u*_min_ = 0, *u*_max_ = 1, *X*_switch_ = 4,000 (*X*_min_ = 3997, *X*_max_ = 4038, 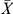 = 4009, *σ* = 11); **E.** *u*_min_ = 0.1, *u*_max_ = 0.9, *X*_switch_ = 4,000 (*X*_min_ = 3998, *X*_max_ = 4578, 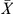 = 4225, *σ* = 214); **F.** *u*_min_ = 0.3, *u*_max_ = 0.7, *X*_switch_ = 4,000 (*X*_min_ = 614, *X*_max_ = 9655, 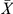 = 4076, *σ* = 2719).

The first feedback rule we investigate, motivated by the structure of optimal solutions to Eq. 14, is to select a critical abundance level *X*_switch_ and define:
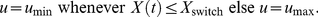
(25)If *X*_switch_ is too large then control is always at its maximum value; as in the case of the baseline values, except *δ* = 7, under seasonal forcing (*d_R_* = 0.45, *d_S_* = 0.45) with *u*_min_ = 0 and *u*_min_ = 1 and *X*_switch_>29,300 (Fig. 3A). As *X*_switch_ is reduced for the baseline set of parameters, control increasingly clips the peaks of the oscillations (Figs. 3B and 3C), thereby reducing the troughs until the consumer population becomes relatively steady around *X*_switch_ = 4,000 (Fig. 3D). The control, however, chatters on-and-off at relatively high frequencies for most of the year, but this can be reduced to chattering for only part of the year if the allocation switching is not complete, but set to *u*_min_ = 0.1 and *u*_min_ = 0.9 (Fig. 3E). If the allocation range is further reduced to to *u*_min_ = 0.3 and *u*_max_ = 0.7 (Fig. 3F), then switching only occurs once to *u*_max_ and once to *u*_min_ each year, but the oscillations in the consumer population again become relatively large.

**Figure 4:**
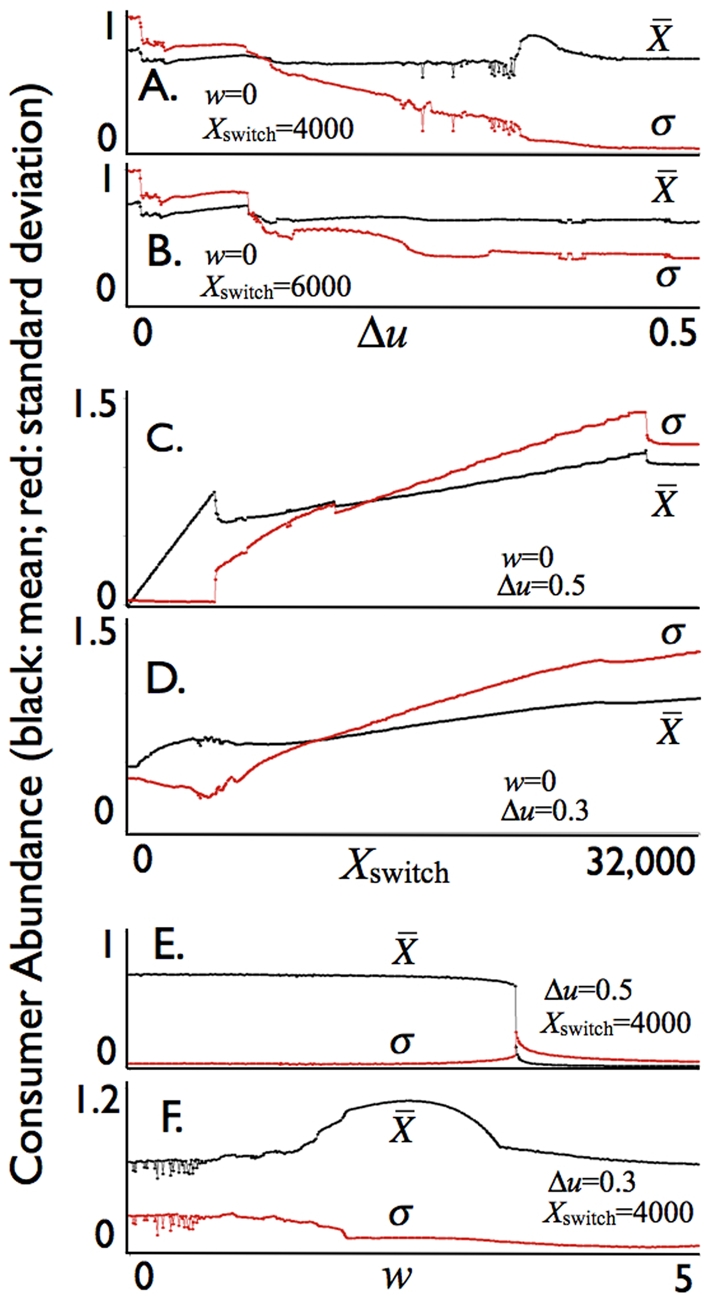
Consumer mean biomass density 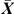 (black: scale 1 = 6,000 units) and its standard deviation *σ* (red: scale 1 = 6,000 units) averaged over a 1000 year period for the allocation parameter *u* switching between *u*_min_ = 0.5-Δ*u* (*X*≤*X*_switch_) and *u*_min_ = 0.5+Δ*u* (*X*>*X*_switch_) with the conversion deviation efficiency cost parameter *w* allowed to vary as indicated. The rest of the parameters are baseline values (Table 2) except that *δ* = 7, *d_R_* = 0.45 and *d_S_* = 0.45, with values for *X*_switch_, Δ*u*, and *w*: **A. & B.** Δ*u* ranging from 0 to 0.5 in steps of 0.001, *v* = 0.5; **C. & D.** *X*_switch_ ranging from 0 to 32000, in steps of 50; **E. & F.** *w* ranging from 0 to 5 in steps of 0.01 (cf. individual trajectories used to obtain the mean and standard deviation for selected values of Δ*u* and *X*_switch_ in Fig. 3, but averaged here over 1000 years to minimize the impact of the initial conditions).

Graphs of the mean and standard deviation of fluctuations in consumer population abundance over a 20-year interval (Fig. 4: years 80–100 are selected from the simulations to avoid transients peculiar to the initial conditions) are plotted over ranges of values for the allocation parameter *u* (Figs. 4A–B), for the threshold parameter *X*_switch_ (Figs. 4C–D), and the loss of efficiency *w* in the deviation of the allocation *u* from the optimal value *v* = 0.70 (rounded to 2 d.p.) (Figs. 4E–F). As the range Δ*u* =  *u*_max_- *u*_min_ increases (cf. Eq. 25), the mean population level 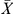 remains relatively constant while the standard deviation *σ* steadily decreases over most of the range (Figs. 4A–B), although small regions do exhibit somewhat irregular behavior due to the highly nonlinear nature of the model. Also in the case *X*_switch_ = 4,000, a favorable region does occur around Δ*u* = 0.35 where the abundance is about 20% higher than for most other values of *u* and the standard deviation takes a noticeable drop down to close to zero for Δ*u*≥0.35 showing the allocation switching rule Eq. 25 is very effective at stabilizing the otherwise strongly oscillatory consumer-resource interaction (cf. Fig. 3A versus 3D).

If *X*_switch_ is too large though, e.g. *X*_switch_ = 6,000, then stabilization is only partial and the standard deviation *σ* remains relatively high over for Δ*u* at its most extreme (i.e. over the range 0.4 to 0.5). This is amply demonstrated in Figs. 4C–D where we see that although 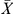 increases with *X*_switch_ the standard deviation *σ* increases much faster once *X*_switch_ gets beyond 4,900, at which point allocation switching looses its ability to completely dampen the oscillations (note, in Fig. 4C, that *σ* is almost zero and then takes a jump around *X*_switch_ = 4,900). The graph in Fig. 4C. indicates a clear advantage for the combination *X*_switch_ = 4,900 and Δ*u*≥0.5 in maximizing the average abundance while completing dampening the oscillations for the baseline set of parameters (though with *δ* = 7 rather than 5).

The graphs in which we vary the efficiency parameter *w* (Figs. 4E–F) indicate that a switching allocation strategy remains very effective even when there is a cost *w*>0 to deviating from the physiologically optimal allocation point of *v* = 0.7 for our baseline parameters (Fig. 1). The stabilization remains relatively insensitive to *w* for the case of extreme switching (Fig. 4E Δ*u* = 0.5) (with mean abundance decreasing only slightly and the standard deviation in this abundance increasing only slightly) until *w* hits a threshold at *w* = 3.4, beyond which the consumer population collapses because it cannot bear the level of cost associated with inefficiencies deviating from the physiologically optimal allocation point *v* = 0.7. Some unexpected happens, however, for the case Δ*u* = 0.3 (Fig. 4F). In this case consumer abundance is maximized, with a corresponding relatively low associated standard deviation, when the population efficiency parameter has the nonzero value *w* = 2.46, implying, as we discuss further below, that some cost to allocation switching is beneficial to the population as a whole.

### Allocation Modes and Growth Patterns

Seasonal growth patterns in which organisms allocate resources to different organ systems—e.g. vegetative structures roots and tubers, or reproductive structures—are well known in plants and animals [65], [66]. The type of tissue laid down in these different organ systems can be viewed as representing different intrinsic quality levels with switches from one growth mode to another subject to resource-demand versus extracted-supply related physiological signals [18]. This sort of growth mode switch does not necessarily require external seasonal signals, but may be linked to signals generated intrinsically through stresses brought about by crowding; with this phenomenon being evident across the organismal spectrum including bacteria, protists, fungi, plants, invertebrates and vertebrates.

Recently [27] presented their deconstruction of what they refer to as a typical growth curve of lab-cultured microbial populations. They identify five to six phases that include (cf. Fig. 1 in [27]): 1.) an initial lag phase in which the culture takes a characteristic “lag time” to begin growing; 2.) a stronger than exponential phase of growth (which they call the logarithmic exponential or LogEx phase); 3.) an exponential phase (which they call the regular exponential or RegEx phase); 4.) an inhibition phase; 5.) a stationary phase; and possibly 6.) a decay/decline phase. They analyze several different classes of models that have been developed to capture all these different phases and they conclude that since “all the theoretical and numerical results presented for delay growth models are contrary to the experimental evidence regarding the conditions for the occurrence of a lag phase, we may conclude that delay effects and the lag are two distinct biological phenomena.” The implication of this is that the lag phenomenon cannot simply be captured through the inclusion of time delays in existing growth models but require an additional dimension to the analysis, such the inclusion of a quality dimension.

**Figure 5:**
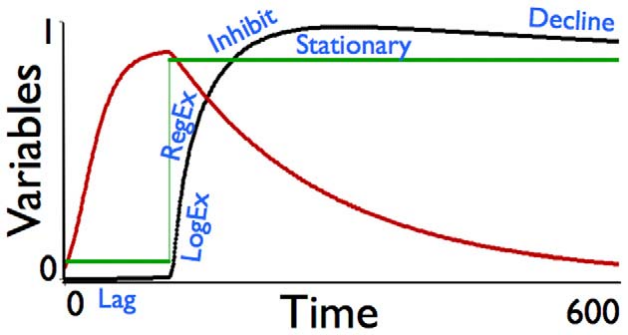
The trajectories of population (consumer) abundance *X*(*t*) (black: scale 1 = 30,000 units; *X*(0) = 100) and quality *Q_X_* (red: scale 0–1; *Q_X_*(0) = 0.05), and the resource extraction allocation function *u*(*t*) (green: scale 0–1) satisfying Eq. 25, with *u*_min_ = 0.07, *u*_max_ = 0.85 and *X*_switch_  = 1000 are plotted over 600 units of time (no longer interpreted as months) under the constant resource conditions *R*(*t*) = 500,000 (instead of Eqs. 17 and 22) and *Q_R_*(*t*) = 0.5 for *t*∈[0,500], for the remaining baseline parameters as in **Table 1** with the exceptions that here *α* = 0.001, *µ* = 0.01, *c* = 0.6, *w* = 1.

These six phases can be captured easily through the allocation switching logic provided by Eq. 25. In Fig. 5 we illustrate these phases for the case *u*_min_ = 0.07, *u*_max_ = 0.85 and *X*_switch_  = 1000. Thus, if the quality and abundance are initially low at the start of a microbial culturing experiment, the population focuses on increasing its quality, thereby producing a lag phase in its growth in abundance until the abundance variable crosses a threshold. Rapid growth is then experienced (LogEx phase), followed by steady growth (RegEx phase), followed by inhibition and then stationarity as density dependence sets in. Note that we could have fashioned the trajectory in Fig. 5 to closely resemble the growth curve idealized in Fig. 1 of [27] by making the allocation switch occur over a finite period of time rather than instantaneously, thereby removing the sharp corner in the abundance trajectory at the switch. The purpose of Fig. 5, however, is merely to demonstrate how allocation switching, whether sharp or gradual, can produce a variety of empirical growth patterns that have been observed in nature or laboratory cultures. One can imagine even more complex growth patterns if switching is based on thresholds in both the quality and abundance rather than just the abundance alone. Finally, due to a decline in quality over the stationary phase, a sample from the population depicted in Fig. 5, if now moved to another culture dish as was done in the experiments described in [27], will exhibit the same pattern of growth because, in the new dish, the initial conditions are now once again low quality and low abundance.

## Discussion

Theoretical population ecology during most of the 20^th^ Century has been developed around an abundance (i.e. quantity) variable involving numbers of individuals, or number or biomass densities. Additionally, populations have been structured into age, size or life-history stage classes ([67], [68], [69]; also see [7], [18], [37], [70]). This is not to say that other subfields of ecology such as physiological and ecosystem ecology have not used other currencies (energy, kinds of molecules, nutrient classes) to discuss individual or community level dynamic processes [71], [72]. In consumer-resource or food web abundance (biomass/numbers-density) dynamics, however, the importance of a second-order description when trying to explain the source of oscillations that are observed in such systems has been largely neglected (to whit see [8]), with the exception being the work of Ginzburg and collaborators [15], [19] and some efforts to include storage as an explicit process [32].

The case for including a second dimension as a necessary step to adequately capturing eigen-frequencies associated with the maternal effects that are characteristic of many consumer-resource interactions (e.g. the 12-generation cycles in vole-lemming interactions as described by Ichausti and Ginzburg [57]) has been most elegantly presented by Ginzburg and Colyvan [15]. Maternal effects may operate among large mammals whereby high-quality mothers produce offspring that are larger at birth and hence have enhanced survival prospects [17]. Shorter time scale inertial processes related to within generation storage, or other within generation structural (e.g. fiber content, body mass) or process-related (e.g. immunology, hibernation) responses, are also likely to play a role as populations, for example, adjust to a period of starvation [73]. With the quality dimensions added to all the species involved in consumer-resource interactions and other more complex trophic cascades [21], [22], [74], it is possible to address evolutionary questions relating to adaptive abundance-quality dynamics as a mechanism for stabilizing population fluctuations, whether induced purely by high growth rates (Fig. 2E) [33], [60], entrained by seasonal drivers (Fig. 2B), or an emergent nonlinear combination of the two (Fig. 2F). Thus our focus is on the questions of what the best allocations of extracted resources may be in increasing the abundance versus the average quality of populations.

From the perspective of long-term population persistence using an autonomous systems (i.e. systems with time-invariant descriptions of how the variables change as a function of their own values) optimization framework, we were able to elucidate how the long-term, physiologically most efficient, allocation proportion v* (Eq. 14) depends on different population parameters. But we were further able to show that adaptive allocation strategies that have evolved to dampen oscillations may deviate from the most efficient, with such deviations being beneficial to the population (Fig. 4F). This counter-intuitive result, after a little thought, actually makes sense. The reason for this has to do with the fact that highly oscillatory behavior emerges in consumer-resource systems when abundance growth rates pass a threshold (cf. Fig. 2A versus 2E). Ginzburg, Burger and Damuth [75] have argued that the “May oscillation threshold” is an upper bound for selection acting to increase growth rates. In a consumer-resource resource context, the destabilization of an interaction that emerges with increases in the efficiency of consumption towards low resource levels is known as the paradox of enrichment. The principle was first graphically explicated by [76], and further investigated in various contexts including that of longer food chains [74] and increased efficiency in converting extracted resources [77]. The simulation outcomes we depict in Figs. 4E and 4F suggest that a physiologically related inefficiency can help mitigate this paradox. Other inefficiencies, or growth rate reducing strategies, such as suboptimal foraging [78], functional heterogeneity in the resources exploited [77], reciprocal phenotypic plasticity between prey and predator species [77], and switching between production of resting versus non-resting eggs [79], have been shown to play this same role. In addition, various species interaction processes including intraspecific competition among consumers [80] and disease [81] stabilize consumer-resource interactions, which in our Q-Q formulation has the added realism of being able to model how these interactive process are impacted by the quality of each population. Our Q-Q formulation also provides ways to incorporate these connections: as we mentioned in developing our quality model Eq. 13, the senescence rate can be elaborated to include increased susceptibility to predation and disease of lower quality individuals.

In our general 4-dimensional consumer-resource formulation (i.e. Eqs. 17–20), as in all differential equation models of population level processes, we have averaged-out the faster behavioral and physiological processes that are relevant to diurnal cycles, while focusing on processes relevant to a seasonal time frame. We have also averaged-out fine scale ecological processes such as birth pulses and extreme weather-related death rates and replaced these with smooth seasonal and quality-related rates. In doing so, a biomass currency is more flexible than a demographic current because biomass growth and decay rates are more continuous over time than changes in numerical abundance, though no system is really continuous at sufficiently fine scales of time: to not recognize this artifact, according to [15], is to fall victim to the fallacy of “instantism”. From a biomass versus numbers point of view, though, births do not entail the production of new biomass (to the contrary some biomass is lost during birth through the shedding of auxiliary structures such as the placenta in mammals), merely the separation of some fraction of maternal biomass in the form of offspring, perhaps provisioned with material resources in the egg or seed, or provided with these after birth by mothers. Additionally, our Q-Q approach allows us to explore seasonal effects on population dynamics that relate to issue of growth as a function of population quality. It is no coincidence that in many populations births tend to be concentrated during the season when resources are high in quality ([39] Chapt. 7, [82]), while most mortality (except among neonates) occurs during adverse periods [83], so that these processes are potentially influenced by different environmental factors. In our model death rates go up as quality goes down (cf. [84]), where quality itself is driven by seasonal factors; but a quality feedback is in place because low quality individuals die or senesce at faster rates.

For purposes of transparency, we have kept the presentation of our Q-Q formulation rather generic: its application to addressing questions relating to specific systems requires closer attention to the rates of the processes involved in such systems than can or should be provided in a presentation of a general framework. Our Q-Q formulation can also be brought to bear on a number of interesting questions, such as the impacts of extreme seasonal variation in environmental conditions on the stability of interacting populations. Previous modeling has suggested that strong seasonality can have a destabilizing effect by promoting oscillations in abundance ([85], [86]; but see [77]). Our Q-Q approach illustrates how this need not be the case, depending among others on the relative values of the time constants associated with resource growth, resource extraction, and responses in consumer quality. If extreme resource deprivation is seasonally predictable, organisms can counteract it by either carrying over stored reserves (body fat in animals, carbohydrate reserves in plants) or by entering dormant stages (as eggs or pupae, or in hibernation) through this period. Hence populations should not be more variable in abundance in higher latitudes despite wider seasonal variation in conditions than in lower latitudes with less extreme winters. Indeed, annual variation in abundance may be greater when seasonal variation is related primarily to rainfall, the typical situation in tropical savannas and grasslands as well as Mediterranean-type systems, and hence is less predictable than when governed mainly by seasonal temperature oscillations [87]. In far northern latitudes, the effect of temperatures exceeding thermal tolerance levels due to global warming may pose less of a threat than that posed by more frequent occurrences of extreme stochastic events, such as cyclones or thaw-freeze alternations [88], [89]. The draw-down in plant biomass and its quality that occurs during the adverse season restricts the resource base from which herbivore biomass density rises during the benign season, and hence the peak seasonal biomass attained by the herbivores ([39]: Chapter 13). More extreme winters mean that there is lesser potential for herbivore populations to grow towards abundance levels depressing vegetation production.

While classical models emphasize the propensity for coupled herbivore-vegetation oscillations to be generated (e.g. [90]), such irruptive dynamics may be less commonly manifested than implied by these models [91]. Preferential consumption of higher-quality vegetation components makes herbivores become more dependent on resources of sufficiently low quality to dampen their growth potential later in the dormant season, which also helps buffer them against population extirpation during severe winters [92]. The cyclic variation in abundance of hares, lynxes, voles and lemmings in far northern environments that has fascinated population ecologists is likewise not widely manifested among similar species elsewhere in the world. The recent fading out of the vole and lemming cycles in Scandinavia [93], [94] presents a fundamental challenge to conventional models that have tried to explain these cycles as being primarily the result of a predator-prey interaction with weasels, without taking into account how the changing effects of winter snow conditions affect the quality as well as the quantity of resources [95]. To incorporate this level of detail requires that we use the full Q-Q formulation represented by Eqs. 17–20, rather than the restricted formulation represented by Eqs. 19-24 (i.e. include a dynamic equation for the variable *Q_R_*). The depression of resource quality as well as quantity has a further influence in suppressing the irruptive potential of herbivore populations. As a result, fluctuations in the abundance of generalist herbivores, such as large ungulates, are likely to be more extreme in fertile environments where more of the plant biomass is highly nutritious than in regions under-laid by nutrient-deficient soils presenting more extreme gradients in the nutritional value of this herbage [77]. Our analysis indicates very clearly, however, that resource extraction switching between increasing the abundance and elevating the quality of consumers provides an extremely powerful mechanism for promoting the stability of consumer-resource interactions.

The overriding importance of variable food quality for population dynamics has been emphasized by [96] for small herbivores and by [97] for large herbivores. Selective grazing can either reduce the effective value of the food resource by promoting the spread of lower-quality plant species or parts, or enhance it by cultivating young growth stages of the plants cropped (e.g. via grazing lawns; [98]). For large carnivores, the effective quality of their food resource depends on the proportion of prey populations in the most vulnerable young or rather aged life history stages [99]. Predation is widely recognized to improve the health (i.e. the effective quality) of prey populations by removing ageing, sick or wounded animals, while the restriction on recruitment can lower the likelihood of the resource base for herbivorous prey becoming over-utilized [100]. For herbivorous insects, poor quality vegetation retards growth through larval stages, thereby increasing vulnerability to mortality from numerous predators [101].

Our Q-Q formulation is easily extended to multi-consumer multi-resource systems, as well as multi-trophic systems. The key to formulating multi-consumer, multi-resource systems developed in the previous sections is through the elaboration of the extraction function given in Eq. 6 to multi-consumer-resource settings, as described in [21], [38]. The key to formulating tritrophic, oligotrophic, or other multi-level food webs comes through the fact that, unlike Lotka-Volterra type equations that use logistic-like growth functions to model resources in the absence of consumers and exponential-like decay functions to model consumers in the absence of resources, our Q-Q formulation is based on a trophic-level-independent equations for both the population quantity and quality variables (Eqs. 11–13). Thus, with appropriate multispecies elaborations (e.g. see [38]) the approach is appropriate for modeling food web interactions in general as they apply to both marine [102], [103] and terrestrial [104] food webs and ecosystems. In the context of food webs, our Q-Q two-state representation of each population facilitates the investigation of the food web dynamics when the condition and health of individuals in populations responds to fluctuations (generally seasonal) in environmental inputs, and changes under the stress of exploitation or diminishing food resources.

### Conclusion

Since the metaphysiological approach provides a unified framework for modeling population interactions at all trophic levels [21], [22], it is not surprising that it extends easily and natural to a Q-Q framework, though inertia associated with the purely abundance approaches is not that easily overcome (cf. [105]). Building on the metaphysiological approach, our Q-Q formulation provides the foundations for models that have the potential to address a number of very broad research questions in ecology. These include a natural way to link physiological and behavioral levels of analysis that depend on quality to population level scales of analyses that are concerned with abundance, while retaining sufficient transparency or simplicity to be able to extract general principles (e.g. in context of plant dispersal see [106]; in the context of resource variability on foraging see [107]; in the context of search and navigation see [108]). In particular, our Q-Q formulation allows scaling to be done through the vehicle of quality as measured by any state feature affecting intrinsic or extrinsic rates of loss relative to resource extraction rates. Thus in scaling up the state of individuals of various types and ages to get a measure of the biomass density of a population in a particular landscape or, more broadly, in a particular ecosystem or subcomponents of that ecosystem, one can also assess the average quality of those individuals and how quality changes with seasons, population density and other relevant factors.

In his recent monograph on population dynamics, Turchin [8] asked a series of questions including “Why do organisms become extremely abundant one year and then apparently disappear a few years later?” He also states “… much progress toward [a general theory of complex population dynamics] has been made.” This may well be true in the context of theories confined to describing each population using a single variable, but the limitation of such theories in addressing questions that incorporate physiological notions of condition, stress, health, climate change and so on are evident. The question Turchin poses goes well beyond purely theoretical interest: it relates very much to species conservation and the ecological factors needed to protect species from extinction under current conditions of global change. We believe that our Q-Q formulation for modeling complex population dynamics provides a much more powerful tool for addressing such questions than paradigms that currently ignore interactions between the quantity and quality aspects of consumers in relation to the quantity and quality of the resources they exploit.

## Methods

The model developed in this paper represents an extension of the metaphysiological approach to population modeled, which is presented in detail elsewhere [21], [22], [31], [38]. Here for the sake of completeness, a quick review of the basic metaphysiological approachis presented using a notation (Table 1) modified to provide greater clarity in developing our two-state Q-Q representation in a multispecies setting.

### One State Representation: Fixed Resource

Let *X* represent the biomass density of a population consuming an underlying resource *R*. We formulate the rate at which a unit of *X* extracts a unit of *R* in terms of an extraction function *f*(*R*,*X*). Using this notation, the total harvest rate experienced by the resource is *f*(*R*,*X*)*X* per unit resource. On the other hand, the total resource extraction (i.e. feeding) rate is *f*(*R*,*X*)*R* per unit consumer. The reason for distinguishing the flow of resources to consumers in terms of a harvest rate versus an extraction rate from the resources will become clear when we develop the model further in a two-species consumer-resource setting.

The gross rate at which a consumer population accumulates biomass per-unit consumer is then the per-unit consumer-biomass feeding rate multiplied by a conversion coefficient *κ* [21], [31], [109]. To get the net per-capita biomass growth rate *g* we need to subtract the metabolic expenditure rate *µ* (i.e. the rate at which organisms dissipate biomass per unit biomass just to maintain physiological processes) to obtain 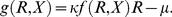
(4)


Since *X* is dynamic, and in a two-species consumer-resource setting *R* is also dynamic, we take advantage of the notational device of a tilde whenever we want to write a function of time-varying variables purely in terms of time *t* itself: that is, we write 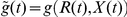. If we use *θ t*o represent a variable extrinsic decay rate per unit *X* (mortality from senescence and disease) and *ε* to represent the extrinsic removal or harvest rate (from predation or simply accidental deaths), then from Eqs. 2–4, dropping the subscript *i*, we obtain the equation
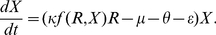
(5)


In the absence of a dynamic relationship between consumers and resource (e.g. if *R* is treated as some constant background level as is implicitly done in writing down a logistic growth equation— for details see [21], [22], [31]), *f* is assumed to decrease with increasing *X*, thereby implying negative density dependence. A function *f* that has the desirable response properties was first proposed by Beddington, DeAngelis and colleagues [110], [111], although the function they proposed is equivalent to our *f*(*R*,*X*)*R*. With this in mind, we adopt their function, though modifying it to include a parameter *γ* that controls the abruptness with which density-dependence sets in [60]. In this case
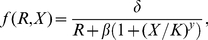
(6)where *δ*>0 is the maximum extraction rate, *β*>0 is the resource level at which the maximum rate drops to half in the absence of interference, and the parameters *K*>0 and *γ*>1 respectively determine the value of *X* around which density-dependence sets in and the abruptness with which it sets in.

In previous formulations of Eq. 5 we assumed, as others also have [21], [31], [33], that *µ* is a constant and *θ* is a function that is inversely proportional to *f*(*R*,*X*). This latter assumption implies that all individuals die as their food intake rate (averaged over the period of time for which this formulation is regarded as applicable) approaches 0. The inverse dependence of *f* on *X* in Eq. 6 and the inverse dependence of *θ* on *f* in the metaphysiological formulation [21], [31] is one way to make the net biomass growth rate *g*, defined by Eq. 4, conform both to the phenomenological criteria of bounded growth and to the empirical observation of accelerating death from starvation (e.g. see description of hydra experiments in [19] or [15]).

### One State Metaphysiological Representation: Consumer-Resource Dynamics

The obvious extension of Eq. 5 to two dimensions is to incorporate the dynamic feedback between the consumer population at abundance (typically biomass density) *X* and its resource base at abundance (biomass or flux density, or concentration as appropriate) *R*. If *R* itself is a functional group of biological populations, such as vegetation in herbivore-vegetation interactions or prey species in prey-predator interactions, then its dynamics will also be governed by a basic metaphysiological equation, but with extraction now explicitly incorporated. In this case both *R* and *X* will satisfy an equation with the structure of Eq. 5, except that for generality in the equation for *R* we use a growth function 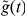, specified generally in terms of time rather than the form given by Eq. 4, and in the equation for *X* we have assumed the consumer itself be free from extrinsic mortality losses (i.e. consumption, predation or harvesting, but subject to senescence only). Thus equations take the form:
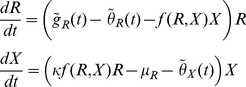
(7)


where our notation makes transparent the way the extraction function *f*(*R*,*X*) ties the consumer and resource equations together [109].

In all our simulations below, we assume that *f*(*R*,*X*) has the form given by Eq. 6. In previous formulations, quality was not considered as a variable so the extrinsic decay rate 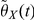 (which includes mortality from senescence, disease, and possibly condition-related predation) was assumed to be proportional to the inverse of the per-capita feeding rate [21], [30], [31], [112]: i.e. for a constant *α*>0 that scales the senescence rate (when feeding or extraction is at is maximum rate *δ* then senescence is at is the minimum rate *α*/*δ*)
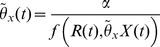
(8)


Although the resource growth and decay process 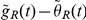 can be modeled using a logistic growth formulation [21], [31], plant biomass growth is generally seasonally phased, with individuals typically ceasing to grow during winter or the dry season, and within-year changes in biomass largely decoupled from inter-annual changes in the plant population components (number of stems or meristems) that produce biomass. The continuous nature of plant biomass extraction by herbivores, however, is compatible with a differential equation expression of this ongoing process, provided seasonal changes in growth are properly taken into account in developing a suitable form for 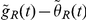. Finally in reconciling Eq. 7 with the first order metaphysiological formulation represented by Eq. 5, we need following identities
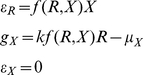
(9)


A controversial question that has been argued back and forth for the past two decades is whether in consumer-resource equations the extraction function *f*(*R*,*X*) should depend on both *R* and *X*, or on *R* only. The question that caused much argument was this: Is the functional response per unit consumer *F* = *f*×*R* as proposed by [113] adequate in assuming dependence on *R* alone, or should it depend more generally on both *R* and *X*, and more specifically on the ratio *R*/*X* ([21], [114]; for a review see [115])? The arguments around this question relate to how we understand the phenomenon of density dependence to emerge through consumer-resource interactions and how this influenced by averaging rates of different time scales [112]. Does it emerge through *interference competition* [116], [117], which affects feeding rates (i.e. *f* is a function of *R* and *X*)? Or, does it emerge as a result of *exploitative competition*, which is indirect since each individual has fewer resources in the future because of greater current resource extraction rates (i.e. *f* is a function of *R* only)? The argument, however, is moot since both interference and exploitative competition operate: only their relative importance is in question and the relative weighting can be expected to vary among populations and possibly between seasons. The short-term processes leading to exploitative competition are often referred to as *scramble competition* [118] while interference competition is also referred to as *contest competition* (e.g. see [119]). In Eq. 6 the value of *γ* in the expression for the feeding function represents the degree to which intraspecific competition is scramble versus contest. Note that *γ* = 0 is the case of pure scramble competition while it has been argued [60] that contest competition requires *γ*>1. The fact that no competition concept falls with the range 0<*γ*<1 and, as argued elsewhere [60], *γ* = 1 is itself problematic strongly suggests that these two types of competition are not opposite ends of the same spectrum, but are fundamentally different types of competition processes that can both operate concurrently, though on different time scales. Scramble competition operates at faster time scales than contest competition, as in the case of territorial animals competing for a resource pulse resulting either from disease, an extreme environmental event (e.g. drought or cold spell: see [120] or carcasses left by hunters or consumers [121]).

## Supporting Information

Appendix S1 Supporting document Appendix S1.
(0.08 MB PDF)

Appendix S2 Supporting document Appendix S2.
(0.07 MB PDF)
