# Supplementary material for: Consumer-Resource Dynamics: Quantity, Quality, and Allocation
Source: PLoS One. 2011 Jan 20;6(1):e14539. doi: 10.1371/journal.pone.0014539 (PMC3024398; doi:10.1371/journal.pone.0014539)
Supplement: Appendix S1 — Supporting document Appendix S1. (0.08 MB PDF) [file pone.0014539.s001.pdf]

Appendices
An Optimality Framework for the Quality of Biological Populations

Wayne M. Getz and Norman Owen-Smith

Appendix S1: Optimal Solutions

Consider the problem:

$$\max_{v,u(t)\in[0,1]} \frac{1}{T}\int_0^T x dt \tag{1}$$

over the period $[0,T]$ for any $T>0$ and subject to equations

$$\frac{dx}{dt} = uI(x,u,v) - \mu + \alpha q, \quad x(0) = x_0, \tag{2}$$

$$\frac{dq}{dt} = -aq(1-u)I(x,u,v) - c\mu - b\alpha q, \quad q(0) = q_0, \tag{3}$$

where in our model

$$I(x,u,v) = \text{sech}\ (w(u(t)-v))\, \frac{\kappa\delta\rho k}{\rho k + k + e^{\gamma x}}.$$

Necessary conditions for $v^* \in [0,1]$ and, for all $t>0$, $u^*(t) \in [0,1]$ to be solutions to this problem is that there exist co-state variables λ_x and λ_q and a Hamiltonian function

$$H(x,q,v,u,\lambda_x,\lambda_q) = \frac{x}{T} + \lambda_x(uI(x,u,v) - \mu + \alpha q) + \lambda_q(-aq(1-u)I(x,u,v) - b\alpha q - c\mu) \tag{4}$$

such that

$$\frac{d\lambda_x}{dt} = -\frac{\partial H}{\partial x} = -\frac{1}{T} - (u\lambda_x - aq(1-u)\lambda_q)\frac{\partial I}{\partial x} \tag{5}$$

$$\frac{d\lambda_q}{dt} = -\frac{\partial H}{\partial q} = -\alpha\lambda_x + (\alpha b + a(1-u)I(x,u,v))\,\lambda_q \tag{6}$$

along solutions x^*, q^*, λ_x^* and λ_q^* generated from Eqs. 2, 3, 5 and 6 using the optimal solutions $v*$ and $u^*(t)$, the condition

$$H\left(x^*,q^*,v^*,u(t)^*,\lambda_x^*,\lambda_q^*\right) \geq H\left(x^*,q^*,v,u(t),\lambda_x^*,\lambda_q^*\right). \tag{7}$$

must be satisfied for all t and all v and $u(t) \in [0,1]$.

Now consider the subclass of problems where we constrain $u(t) = v$ for all $t \geq 0$ and are free to select x_0 and q_0 such that the system is at equilibrium for our various choices of v. What now is the optimal value v^* for this restricted problem. Under noting that when $u = v$, $I(x,v,v)$ is reduced to the following function that is now independent of v:

$$I(x) = \frac{\kappa\delta\rho k}{\rho k + k + e^{\gamma x}} \;\Rightarrow\; e^{\gamma x} = \frac{\kappa\delta\rho k}{I(x)} - (\rho k + k) \tag{8}$$

From this we note that

$$\frac{\partial I}{\partial x} = -\frac{\kappa\delta\rho k\gamma e^{\gamma x}}{(\rho k + k + e^{\gamma x})^2} = -\gamma I(x) + \frac{\gamma(\rho k + k)}{\kappa\delta\rho k\gamma} I(x)^2 \tag{9}$$

Applying the necessary conditions under equilibrium conditions and the constraint $u(t) = v^*$, then using Eq. 9 and the notation $I^* = I(x^*)$, Eqs 2, 3, 5 and 6 respectively reduce to

$$v^* I^* - \mu + \alpha q^* = 0 \tag{10}$$

$$-aq^*(1 - v^*)I^* - b\alpha q^* - c\mu = 0 \tag{11}$$

$$\frac{1}{T} + \left(v^*\lambda_x^* - aq^*(1 - v^*)\lambda_q^*\right)\left(-\gamma I^* + \frac{\gamma(\rho k + k)}{\kappa\delta\rho k\gamma} I^{*2}\right) = 0 \tag{12}$$

$$\alpha\lambda_x^* - \left(b\alpha + a(1 - v^*)I^*\right)\lambda_q^* = 0 \tag{13}$$

Also, under the constraint that $u(t) = v$ for all $t \geq 0$, whenever $v^* \in (0, 1)$, Eq. 7 implies that

$$\left.\frac{\partial H}{\partial v}\right|_{x^*, q^*, v^*, v^*, \lambda_x^*, \lambda_q^*} = 0 \;\Rightarrow\; \left(\lambda_x^* + aq^*\lambda_q^*\right) I^* = 0. \tag{14}$$

Equation 10 implies

$$I^* = \frac{\mu - \alpha q^*}{v^*} \tag{15}$$

and, since $I^* \neq 0$, Eq. 14 implies

$$\lambda_x^* = -aq^*\lambda_q^*.$$

Substituting this last expression in Eq. 13 we obtain

$$\left(-\alpha a q^* - \left(b\alpha + a(1 - v^*)I^*\right)\right)\lambda_q^*$$

Thus either $\lambda_x^* = \lambda_q^* = 0$ or

$$q^* = -\frac{b\alpha + a(1 - v^*)I^*}{\alpha a}. \tag{16}$$

Eqs. 15 and 16 can be simultaneously solved to express I^* and q^* in terms of v^* to obtain:

$$I^* = \frac{a\mu + \alpha b}{a(2v^* - 1)} \tag{17}$$

and

$$q^* = -\frac{\alpha b v^* + a\mu(1 - v^*)}{a\alpha(2v^* - 1)} \tag{18}$$

The relationships in Eq. 17 and 18 can be substituted into Eq. 11 and then solved for v^* to obtain

$$v^* = \frac{a\mu\big(\alpha(b + c) - a\mu\big) \pm \sqrt{ac\alpha\mu(\alpha b + a\mu)^2}}{4\alpha ac - (\alpha b - a\mu)^2}.$$
