# Supplementary material for: Consumer-Resource Dynamics: Quantity, Quality, and Allocation
Source: PLoS One. 2011 Jan 20;6(1):e14539. doi: 10.1371/journal.pone.0014539 (PMC3024398; doi:10.1371/journal.pone.0014539)
Supplement: Appendix S2 — Supporting document Appendix S2. (0.07 MB PDF) [file pone.0014539.s002.pdf]

Appendix S2: Stability of Equilibria

Here we consider the stability of equilibrium solutions to **Main Text Eqs. 11-13** for the case $u(t)$ a constant with $u = v$. To simplify then notation we set $s = k(\rho + 1)$ and $r = a(1 - u)/u$ (note we have excluded $u = 0$) and rescale all time constants in units of time that correspond to $u\kappa\delta\rho k = 1$. In this case the equations reduce to:

$$\frac{dx}{dt} = \frac{1}{s + e^{\gamma x}} - \mu + \alpha q, \tag{19}$$

$$\frac{dq}{dt} = -\frac{rq}{s + e^{\gamma x}} - c\mu - b\alpha q. \tag{20}$$

First we note that since $s > 0$, $r > 0$, $b > 0$, $c > 0$, $k > 0$, $\alpha > 0$, $\mu > 0$ and $\gamma \geq 1$, it follows that $q(t)$ is bounded above by 0 (since $\frac{dq}{dt}|_{q=0} = -c\mu < 0$). Thus we are interested in equilibrium solutions $(\hat{x}, \hat{q})$ for which $\hat{q} \leq 0$ and the corresponding equilibrium total mortality rate on x is

$$\hat{m} = \mu - \alpha\hat{q} > 0, \tag{21}$$

from which it follows that an equilibrium solution $\hat{x}$ exists and is given by

$$\frac{1}{s + e^{\gamma\hat{x}}} = \mu - \alpha\hat{q} \;\Rightarrow\; e^{\gamma\hat{x}} = \frac{1}{\mu - \alpha\hat{q}} - s \;\Rightarrow\; \hat{x} = \frac{1}{\gamma}\ln\left(\frac{1}{\mu - \alpha\hat{q}} - s\right) \text{ provided } \mu - \alpha\hat{q} > \frac{1}{s}. \tag{22}$$

If we now set the rhs of Eq. 20 to zero and substitute the first expression in Eq. 22, we obtain the quadratic equation in $\hat{q}$

$$r\alpha\hat{q}^2 - (r\mu + b\alpha)\hat{q} - c\mu = 0 \tag{23}$$

that has the two solutions

$$\hat{q}_{\pm} = \frac{(\alpha b + \mu r) \pm \sqrt{4\alpha\mu cr + (\alpha b + \mu r)^2}}{2\alpha r}. \tag{24}$$

Since all parameters are positive, $q_+ > 0$ and $q_- < 0$. Thus the latter is the only one applicable to our analysis and below when we use the notation $\hat{q}$ then $\hat{q}_-$ is implied and $\hat{x}$ is the corresponding value of x obtain using Eq. 22.

Linearizing Eqs. 19 and 20 around an applicable equilibrium $(\hat{x}, \hat{q})$, we obtain the Jacobian stability matrix

$$J(x,q) = \begin{pmatrix} \frac{-\gamma e^{\gamma x}}{(s+e^{\gamma x})^2} & \alpha \\ \frac{\gamma e^{\gamma x} rq}{(s+e^{\gamma x})^2} & \frac{-r}{s+e^{\gamma x}} - b\alpha \end{pmatrix}.$$

Noting from Eqs. 21 and 22 that

$$\hat{q} = \frac{\mu - \hat{m}}{\alpha} \quad \text{and} \quad \frac{e^{\gamma\hat{x}}}{(s + e^{\gamma\hat{x}})^2} = \hat{m}\,(1 - s\hat{m}) \tag{25}$$

with a solution existing provided

$$s\hat{m} - 1 > 0 \tag{26}$$

it follows that

$$J(\hat{x},\hat{q}) = \begin{pmatrix} \gamma\hat{m}\,(s\hat{m}-1)) & \alpha \\ \gamma r(\hat{m}-\mu)\hat{m}\,(s\hat{m}-1)\,/\alpha & -r\hat{m}-b\alpha \end{pmatrix}$$

The eigenvalues of $\hat{J}$ are

$$\hat{\lambda}_{\pm} = \frac{\mathrm{tr}\,\hat{J} \pm \sqrt{(\mathrm{tr}\,\hat{J})^2 - 4\det\hat{J}}}{2}$$

where

$$\mathrm{tr}\,\hat{J} = -b\alpha - (\gamma + r)\hat{m} + \gamma s\hat{m}^2.$$

Since all the parameters are positive it follows from Eqs. 21 (recalling $\hat{q} < 0$) and 26 that

$$\det\hat{J} = \gamma(s\hat{m}-1)(\alpha b + r\hat{m} + r(\hat{m}-\mu)) > 0.$$

Thus the eigenvalues are real and at least one is negative. Thus the equilibrium $(\hat{x},\hat{q})$ is either a stable node or saddle point, thereby excluding local oscillations in arbitrary small neighborhoods of the solution $(\hat{x},\hat{q})$.
